# Supplementary material for: High-resolution reconstruction of a Jumbo-bacteriophage infecting capsulated bacteria using hyperbranched tail fibers
Source: Nat Commun. 2022 Nov 24;13:7241. doi: 10.1038/s41467-022-34972-5 (PMC9700779; doi:10.1038/s41467-022-34972-5)
Supplement: Supplementary file 1 — Supplementary Information [file 41467_2022_34972_MOESM1_ESM.pdf]

Supplementary

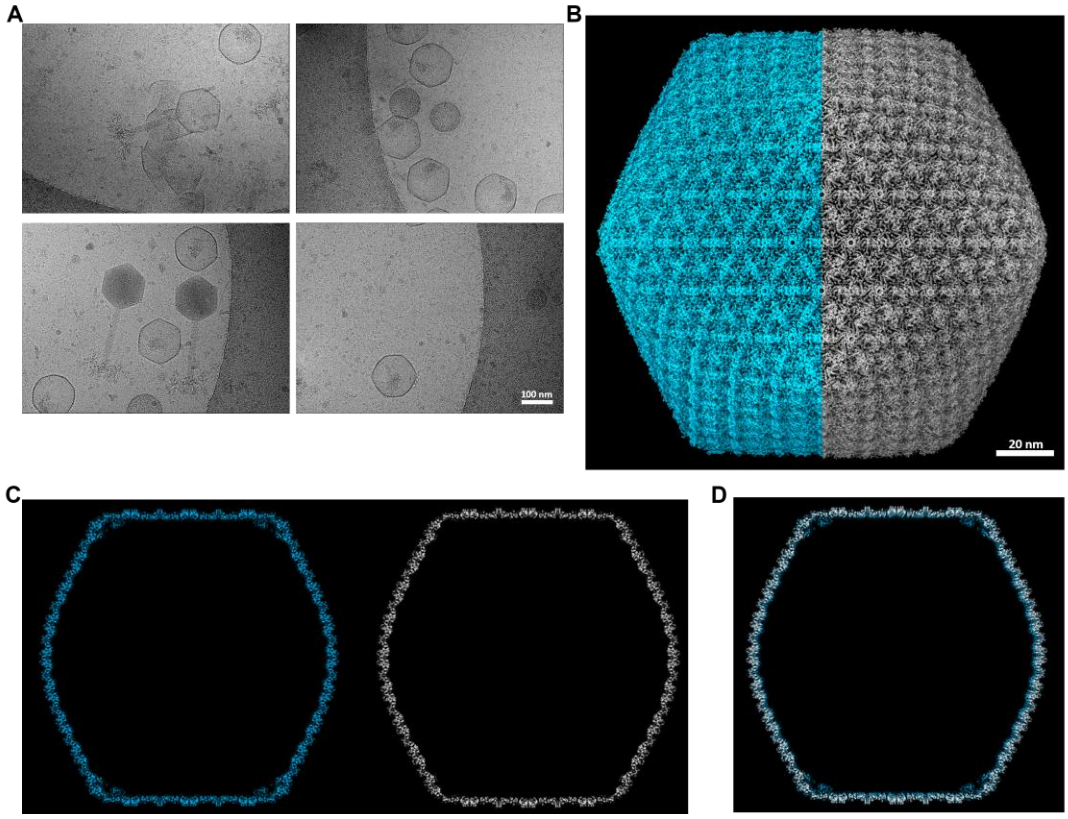

Supplementary Fig. 1 | Capsid comparison. (A) 4 representative micrographs of phage ϕKp24, selected from 34,229 micrographs. **(B)** The comparison of phage ϕKp24 full capsid (EMD-14356, cyan) and empty capsid (gray) showed in ChimeraX. The two capsids use the same organization. **(C)** Central cross-section of full capsid (cyan) and empty capsid (gray) viewed along an icosahedral two-fold axis. **(D)** The overlay view of two central cross-sections showed in **C**. The full capsid (cyan) is a little smaller than empty capsid (gray), which means the full-DNA capsid is more compact than the empty one. The diameter of the full capsid is about 8 Å shorter than the diameter of the empty capsid from vertex to vertex, and about 12 Å shorter along the 2-fold symmetry axis. The DNA is tightly packed inside the full capsid. Additional density is present inside the capsid under the five-fold vertices. However, the major capsid proteins are insufficient in size to account for these densities, and therefore we hypothesize that they arise from internal decoration.

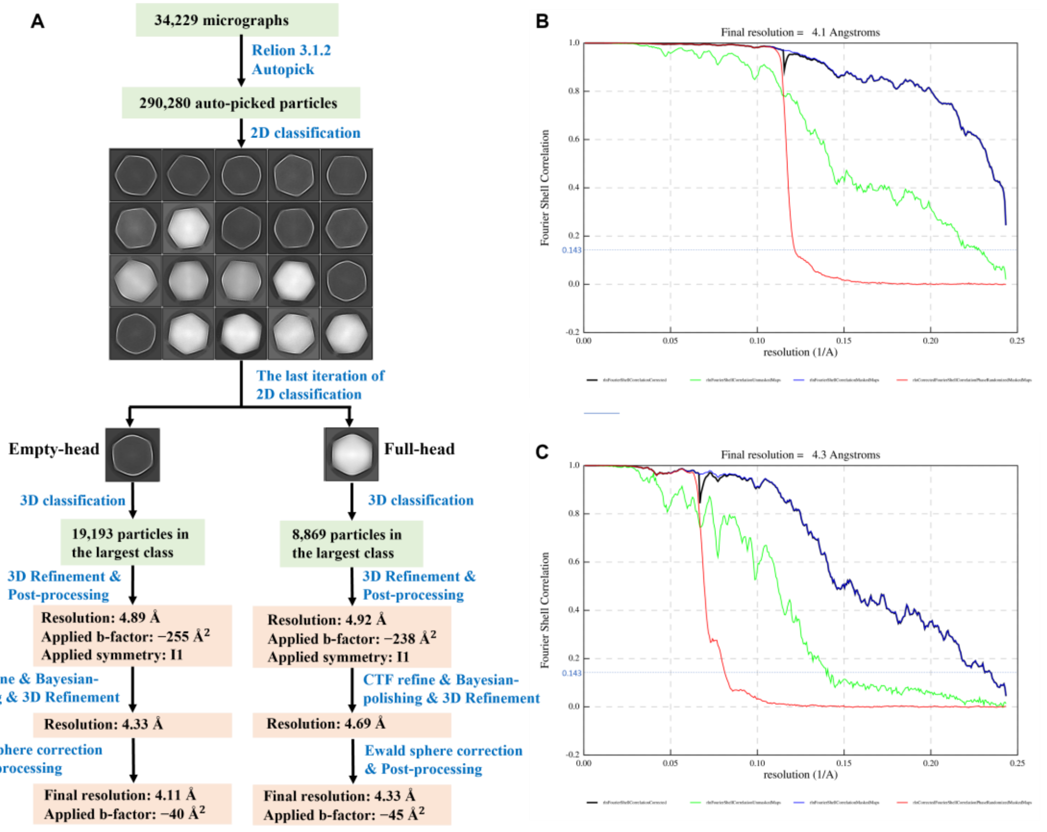

Supplementary Fig. 2 | Reconstruction of capsid. (A) Workflow for the cryo-EM 3D reconstruction of both empty capsid and full capsid of Phage ϕKp24. The pixel size of particles is 2.055 Å, the box size of representative 2D classes is 800 pixels, and the mask diameter is 1600 Å. **(B)** The empty capsid was reconstructed to 4.1Å resolution which is the highest-achievable resolution corresponding to the pixel size (2.055 Å). **(C)** The "gold-standard" $FSC_{0.143}$ criterion shows the full capsid was reconstructed to 4.3Å resolution.

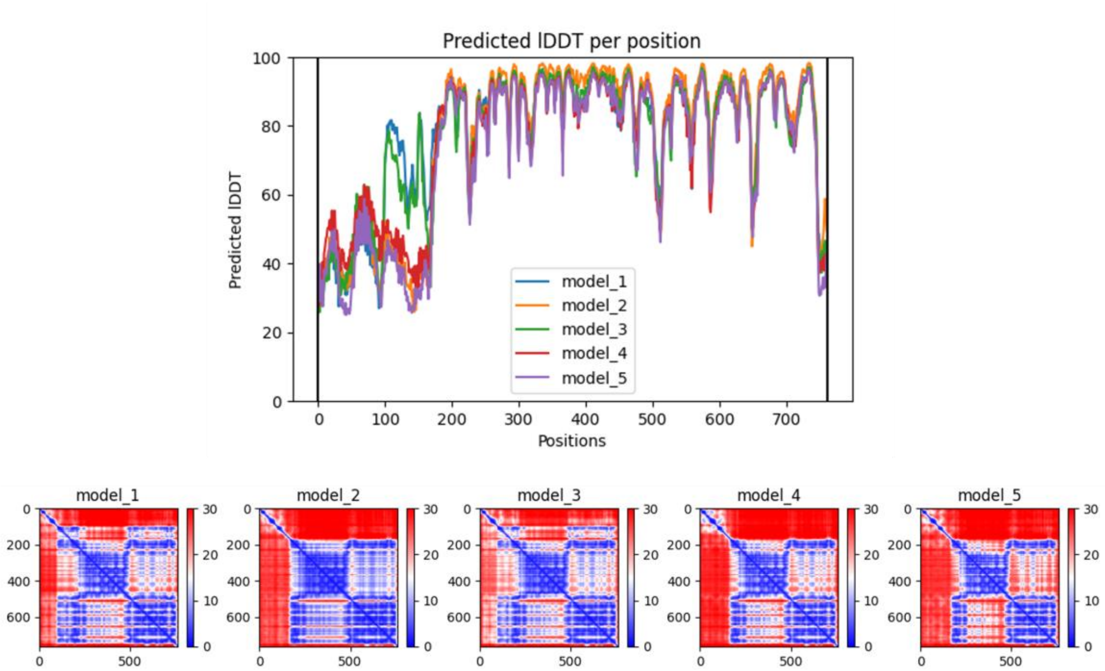

Supplementary Fig. 3 | The predicted IDDT of ϕKp24's MCP gp372, generated from AlphaFold2.

The model 1 was used for model building.

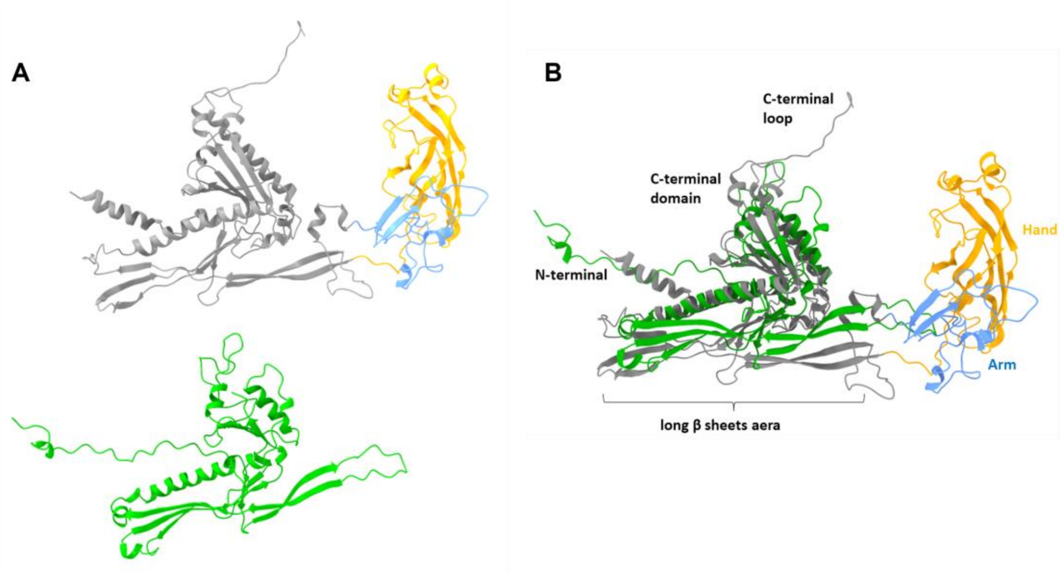

Supplementary Fig. 4 | Comparison of gp372 and MCP of HK97 (A) A separated view of the comparison between phage ϕKp24’s MCP gp372 (gray, blue, orange) and the MCP (green) of HK97, using Matchmaker for alignment in ChimeraX. **(B)** The triangle-part (gray) of Phage ϕKp24’s MCP gp372 compared with MCP (green) of HK97. The triangle-part shows a similar core fold as same as phage HK97, except the long C-terminal loop, the N-terminal and C-terminal domain. The size of long β sheets area is different.

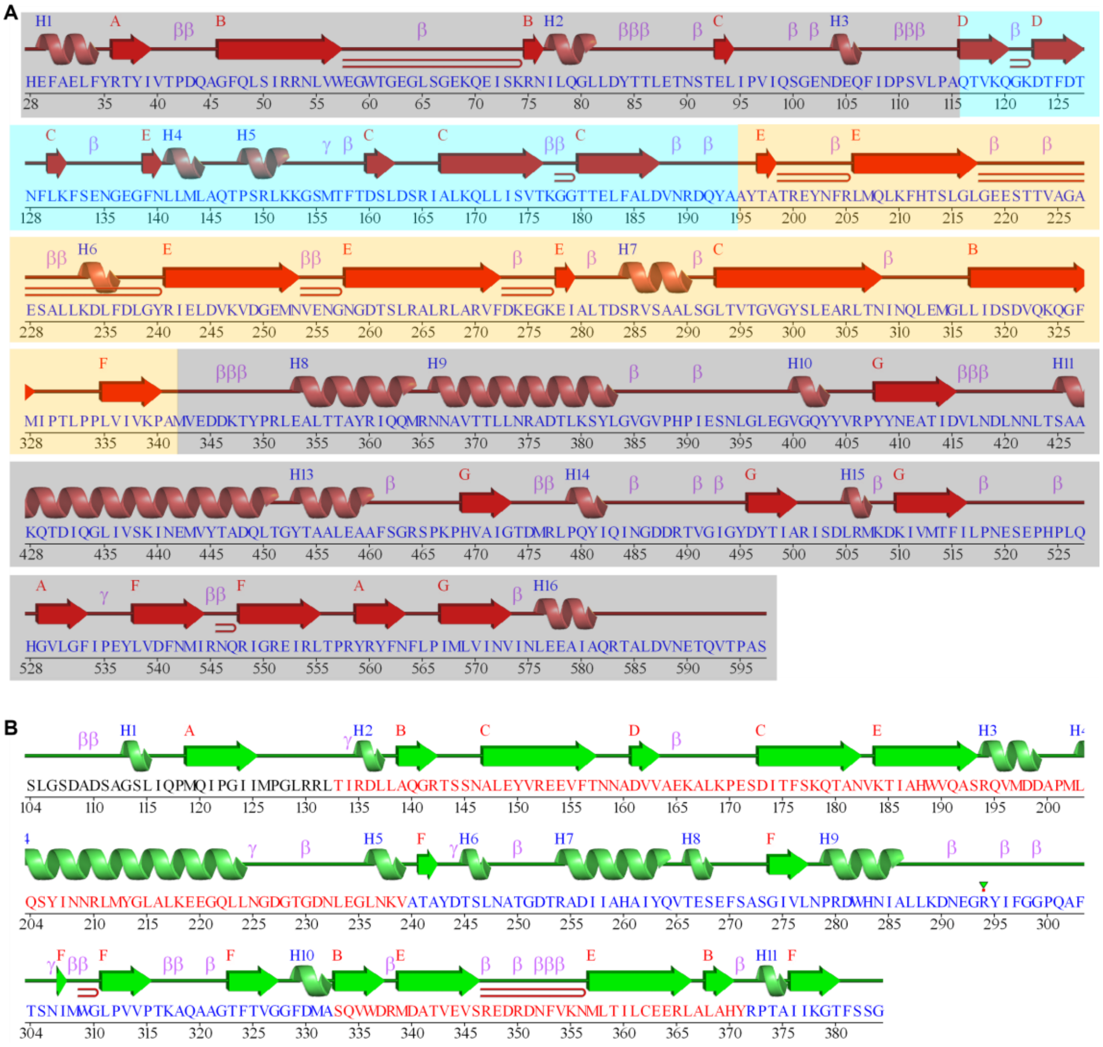

Supplementary Fig. 5 | The secondary structure diagrams of gp372 and MCP of HK97 (A) The diagram of phage ϕKp24's MCP gp372 ("triangular body", grey; "arm", cyan; and "hand", orange). **(B)** The diagram of phage HK97's MCP gp5 (PDB ID: 1FH6).

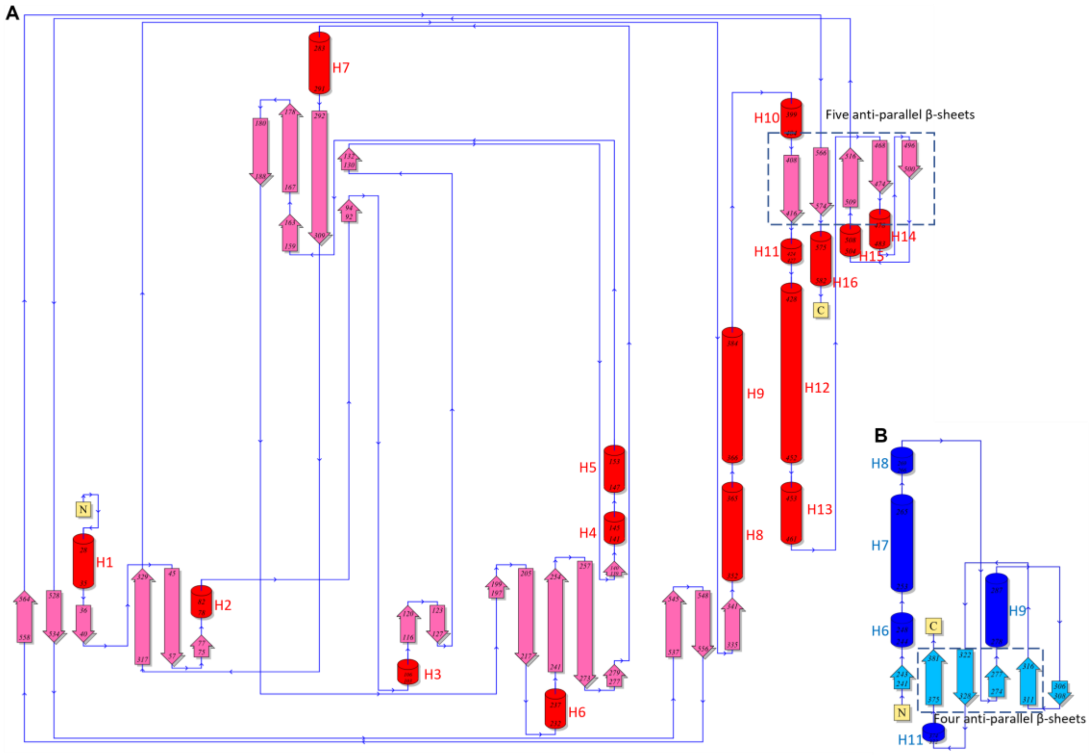

Supplementary Fig. 6 | The 2D secondary structure diagrams (topology) of gp372 and MCP of HK97 (A) The topological diagram (red) of phage ϕKp24's MCP gp372. **(B)** The topological diagram (blue) of HK97's MCP gp5 domain 2.

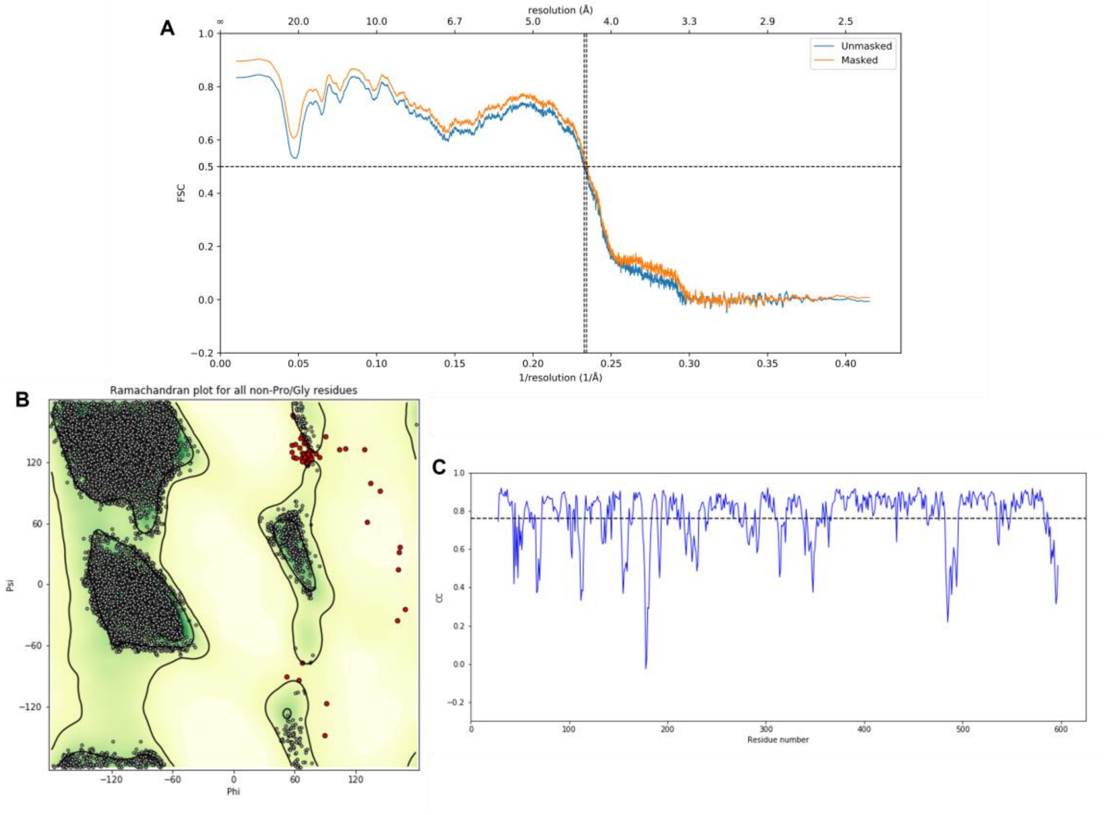

Supplementary Fig. 7 | Validation of the hexamer model (A) Model-map FSC curve of the hexamers model (1 central hexamer, 6 hexamers). **(B)** Ramachandran plot for all residues. **(C)** CC curve for all residues of one major capsid protein gp372.

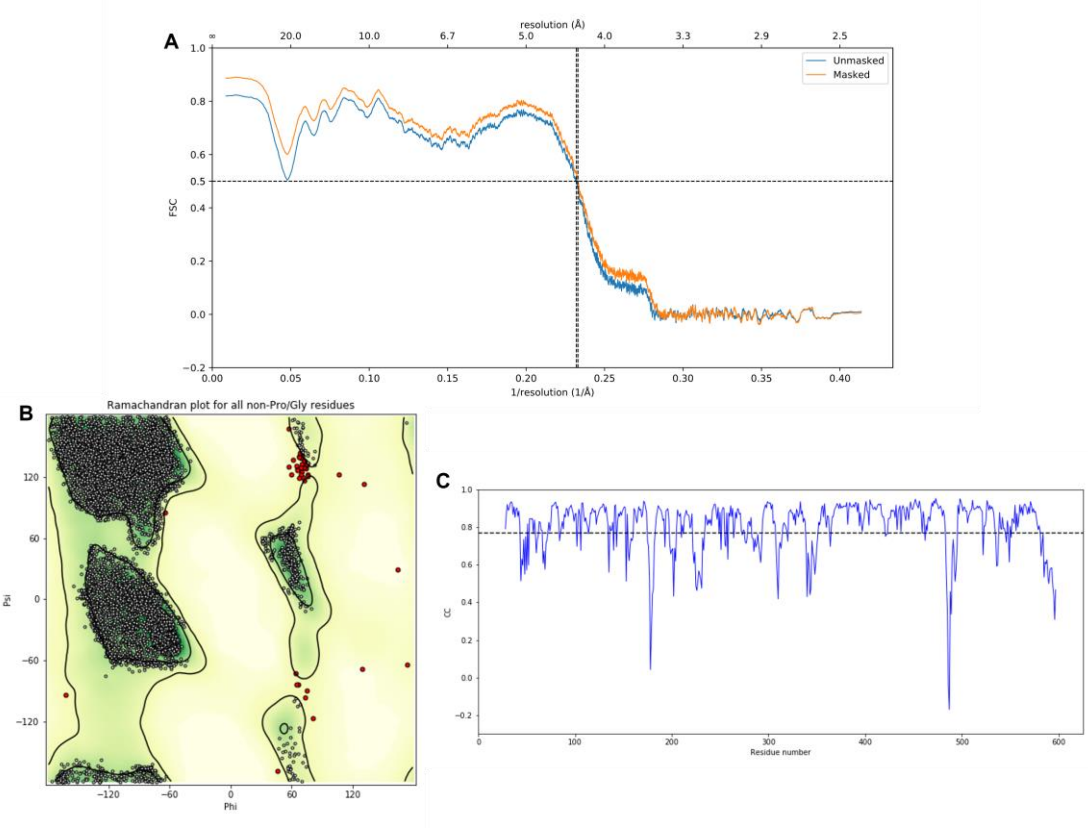

Supplementary Fig. 8 | Validation of the pentamer model (A) Model-map FSC curve of the pentamer model (1 central pentamer, 5 hexamers). **(B)** Ramachandran plot for all residues. **(C)** CC curve for all residues of one major capsid protein gp372.

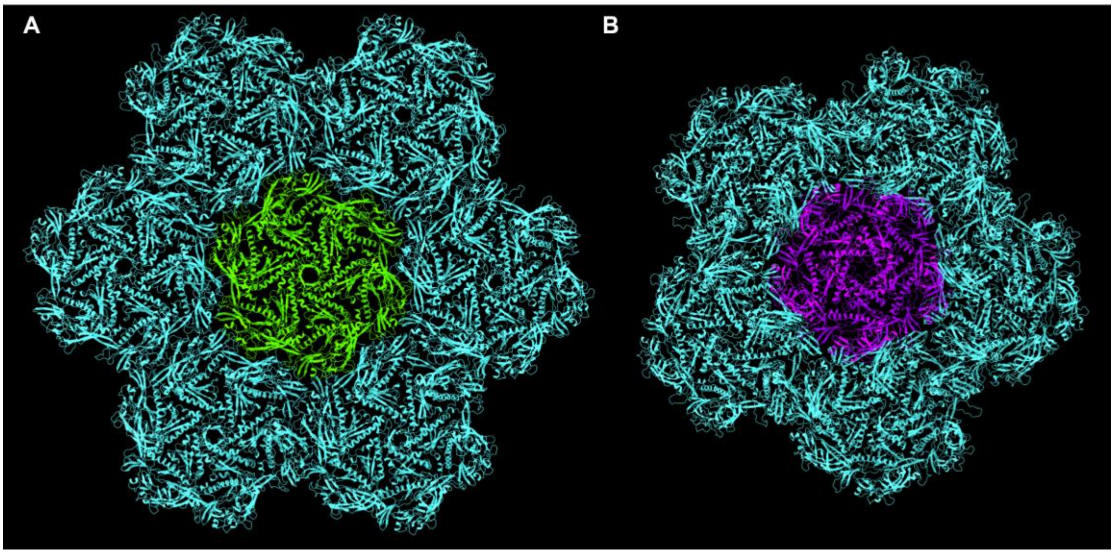

Hexamer contacts					
Triangle-triangle interactions, CW neighbor, same hexamer		Triangle-triangle interactions, CCW neighbor, same hexamer		HAND-HAND interactions, neighboring hexamer	
LYS,380	GLU,220	ASP,163	ARG,558	GLU,201	LYS,210
GLU,398	ARG,190	ARG,190	GLU,398	LYS,210	GLU,201
ARG,477	GLU,442	GLU,220	LYS,380	LYS,210	GLU,251
ARG,554	GLU,313	GLU,313	ARG,554	LYS,247	ASP,260
ARG,558	ASP,163	ASP,419	ARG,583	ASP,249	ARG,264
ARG,583	ASP,419	GLU,442	ARG,477	GLU,251	LYS,210
ARG,583	ASP,587	ASP,587	ARG,583	GLU,251	ARG,264
				ASP,260	LYS,247
				ASP,260	ARG,264
				ARG,264	ASP,249
				ARG,264	GLU,251
				ARG,264	ASP,260

Pentamer contacts					
Triangle-triangle interactions, CW neighbor, same pentamer		Triangle-triangle interactions, CCW neighbor, same pentamer		HAND-HAND interactions, neighboring hexamer	
LYS,380	GLU,220	ASP,163	ARG,558	GLU,201	LYS,210
ARG,501	ASP,448	GLU,220	LYS,380	LYS,210	GLU,201
ARG,558	ASP,163	GLU,442	ARG,583	LYS,210	GLU,251
ARG,583	GLU,442	ASP,448	ARG,50	LYS,247	ASP,260
				ASP,249	ARG,264
				GLU,251	LYS,210
				ASP,260	LYS,247
				ARG,264	ASP,249

Supplementary Fig. 9 | Hexamer and pentamer models, and putative interactive contacts (A) A hexamer surrounding with six hexamers. One hexamer consists of six gp372 proteins and the model was

built by the MDFF method. The center hexamer was colored in green, other hexamers were colored in cyan. **(B)** A pentamer surrounding with five hexamers. Pentamer consists of five gp372 proteins. The center pentamer was colored in purple and hexamers were colored in cyan. **(Tables)** the lists of the putative inter-MCP contacts for hexamer and pentamer.

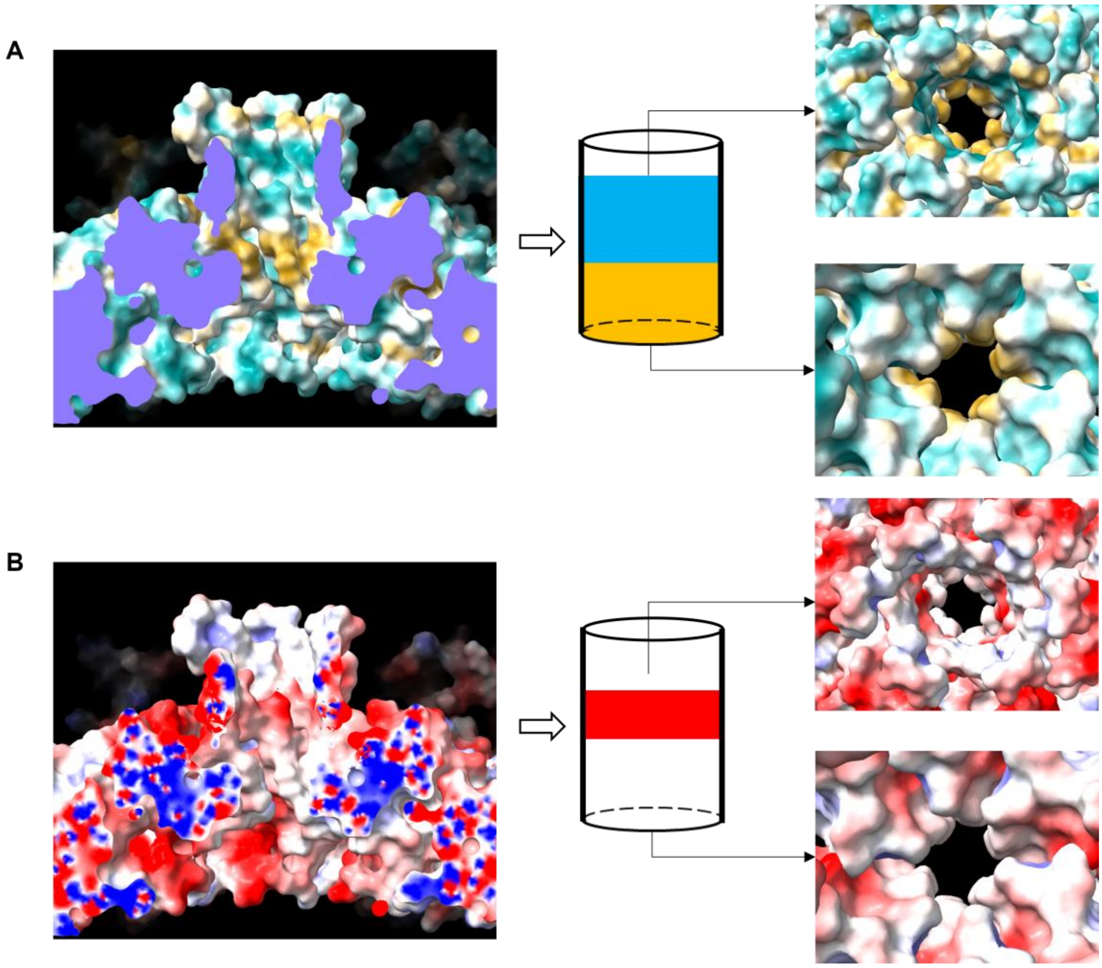

Supplementary Fig. 10 | Properties of the pore's inner surface (A) Hydrophobic surface of the pore (side-cut view, schematic diagram, top view, and bottom view) shown in ChimeraX. The results are shown with coloring on the molecular surface ranging from dark cyan (most hydrophilic) to white to gold (most lipophilic). **(B)** Electrostatic potential of the pore (side-cut view, schematic diagram, top view, and bottom view). Charge distribution: red = negative; blue = positive; white = neutral.

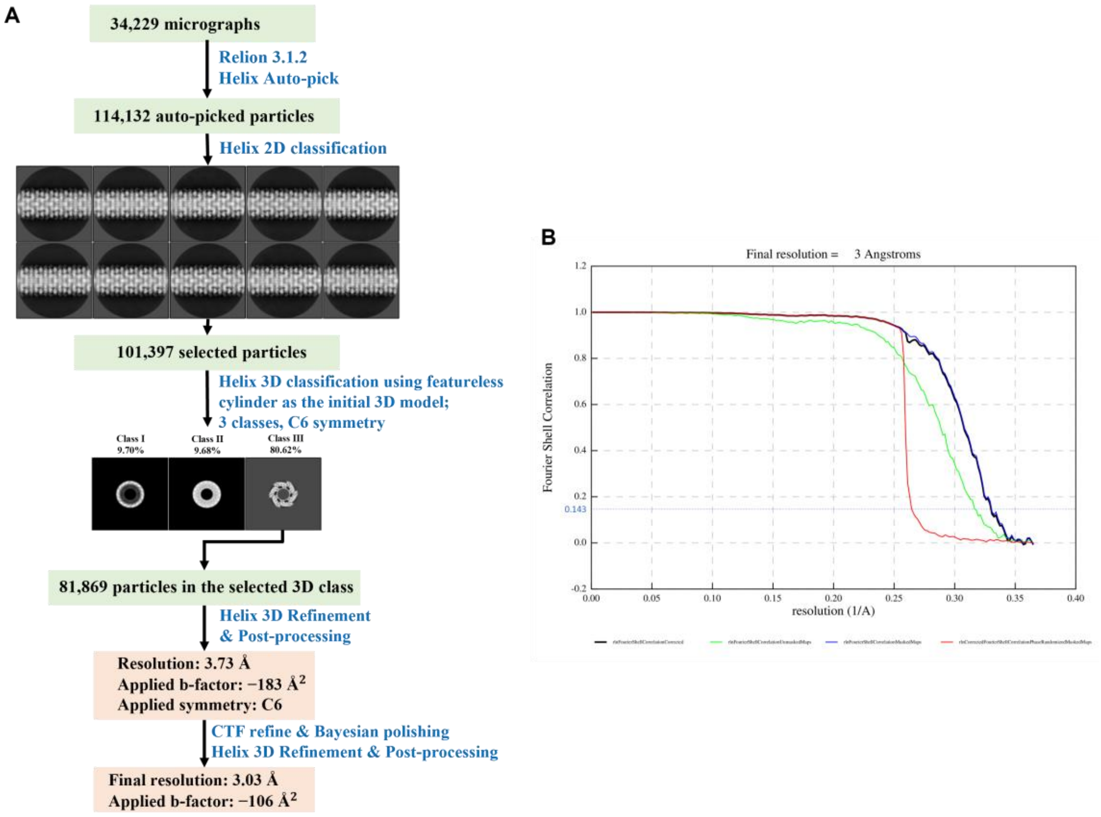

Supplementary Fig. 11 | Helical reconstruction of tail (A) Workflow for the cryo-EM reconstruction of helical tail structure. The box size of representative 2D classes is 450 pixels, the mask diameter is 600 Å. **(B)** The "gold-standard" $FSC_{0.143}$ criterion shows the helical tail structure in its extended conformation has been determined to a resolution of 3.0 Å.

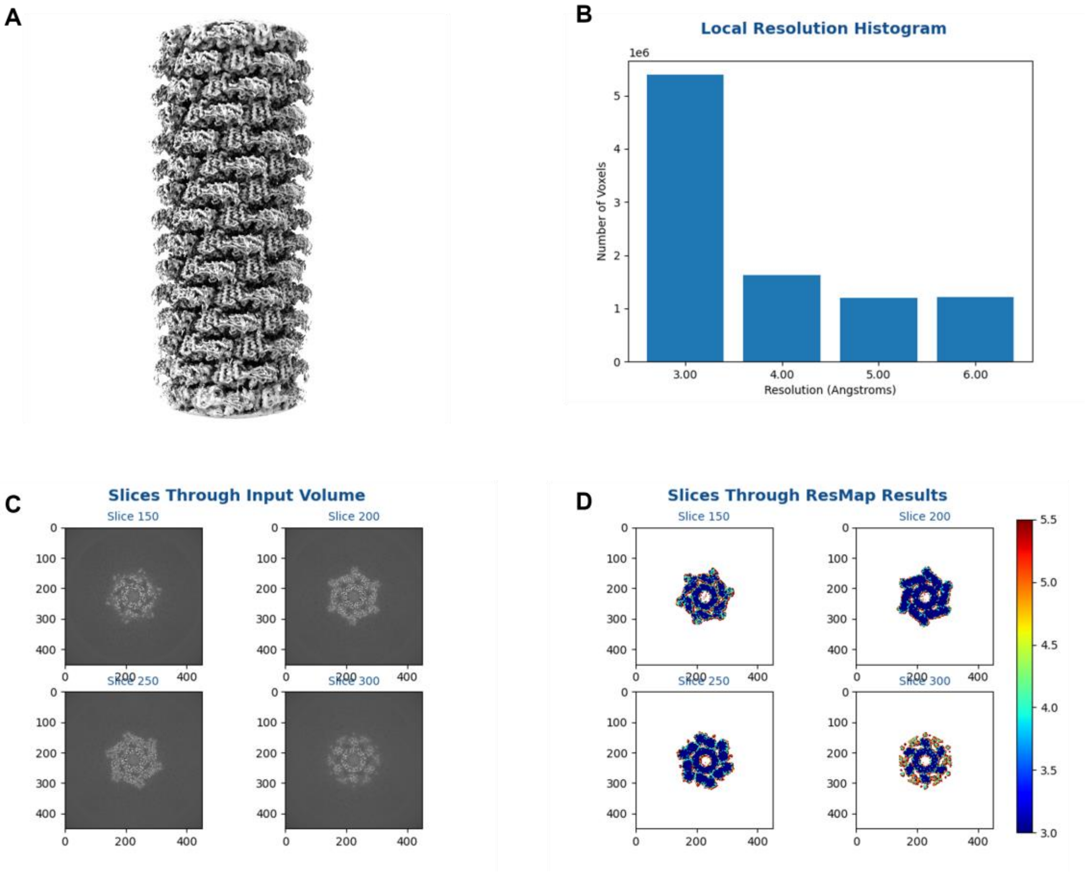

Supplementary Fig. 12 | Local resolution of the extended tail (A) Side view of the helical reconstructed tail showed in ChimeraX, box size is 450 pixels (length is 61.65 nm). **(B)** ResMap results of local resolution histogram. **(C)** The slices through input volume. **(D)** The slices through ResMap results, which shows most of the helical tail structure in its extended conformation has been determined to a resolution of 3.0 Å.

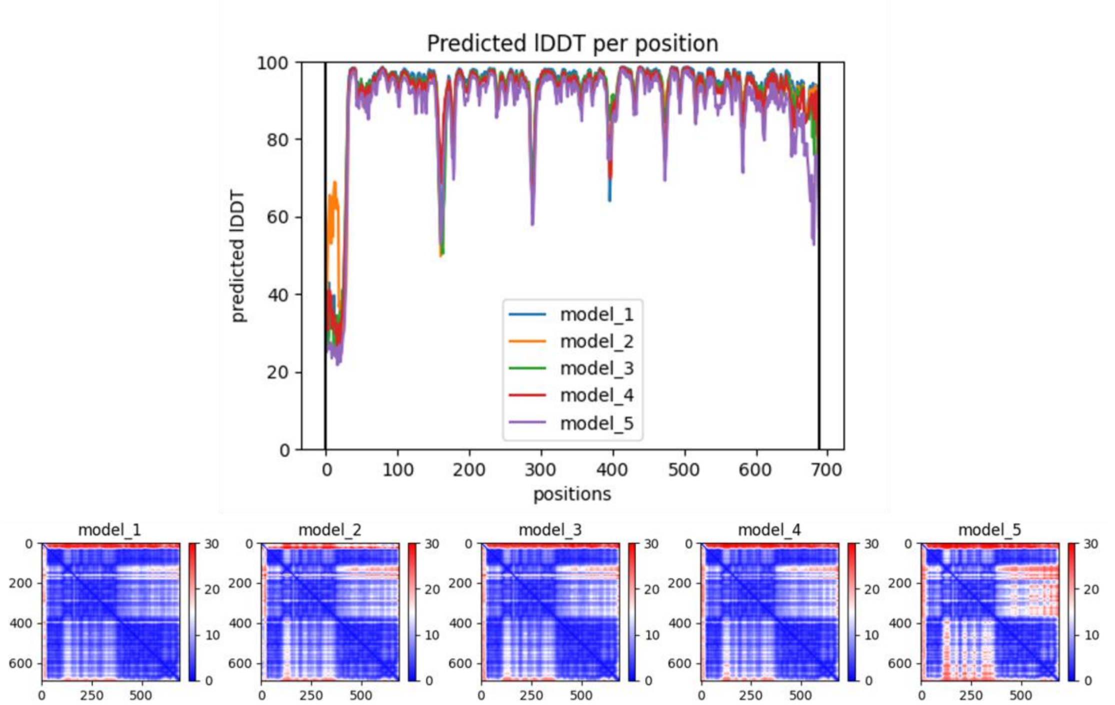

Supplementary Fig. 13 | The predicted IDDT of ϕKp24's sheath protein gp118, generated from AlphaFold2. The model 1 was used for model building.

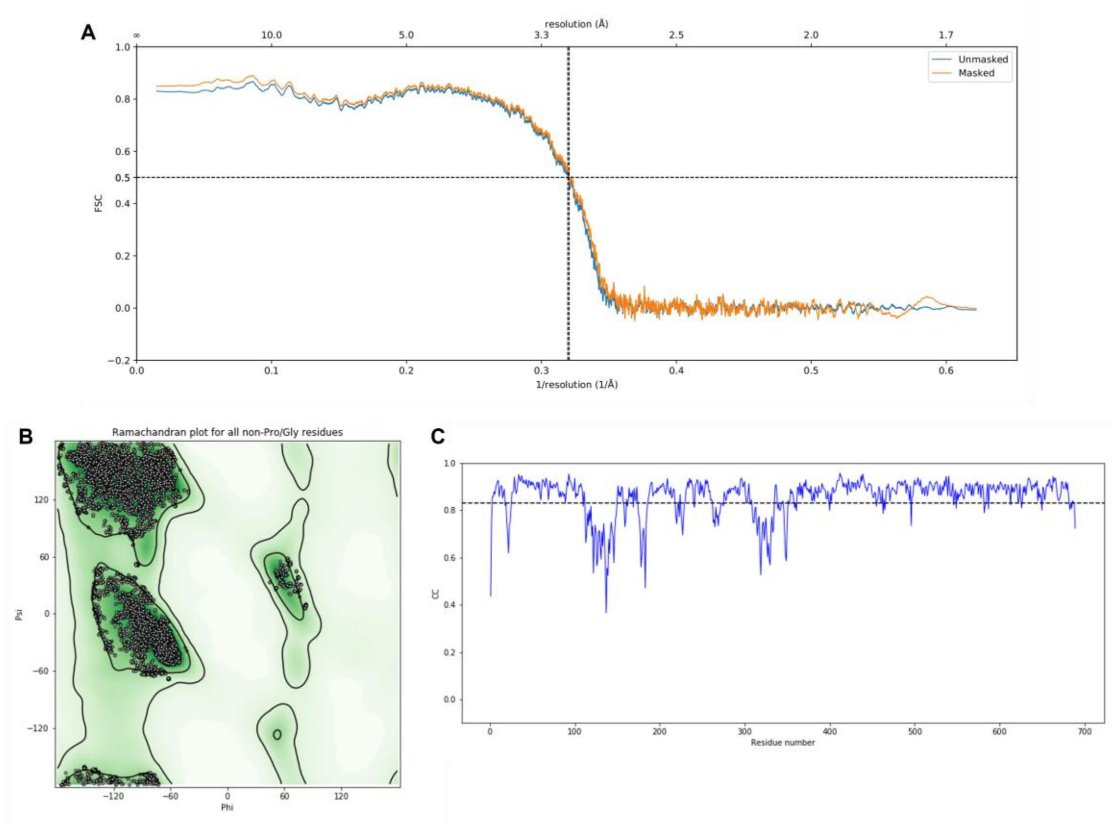

Supplementary Fig. 14 | Validation of the Outer sheath model (A) Model-map FSC curve of outer sheath (3 discs, 18 monomers). **(B)** Ramachandran plot for all residues. **(C)** CC curve for all residues of one sheath monomer gp118.

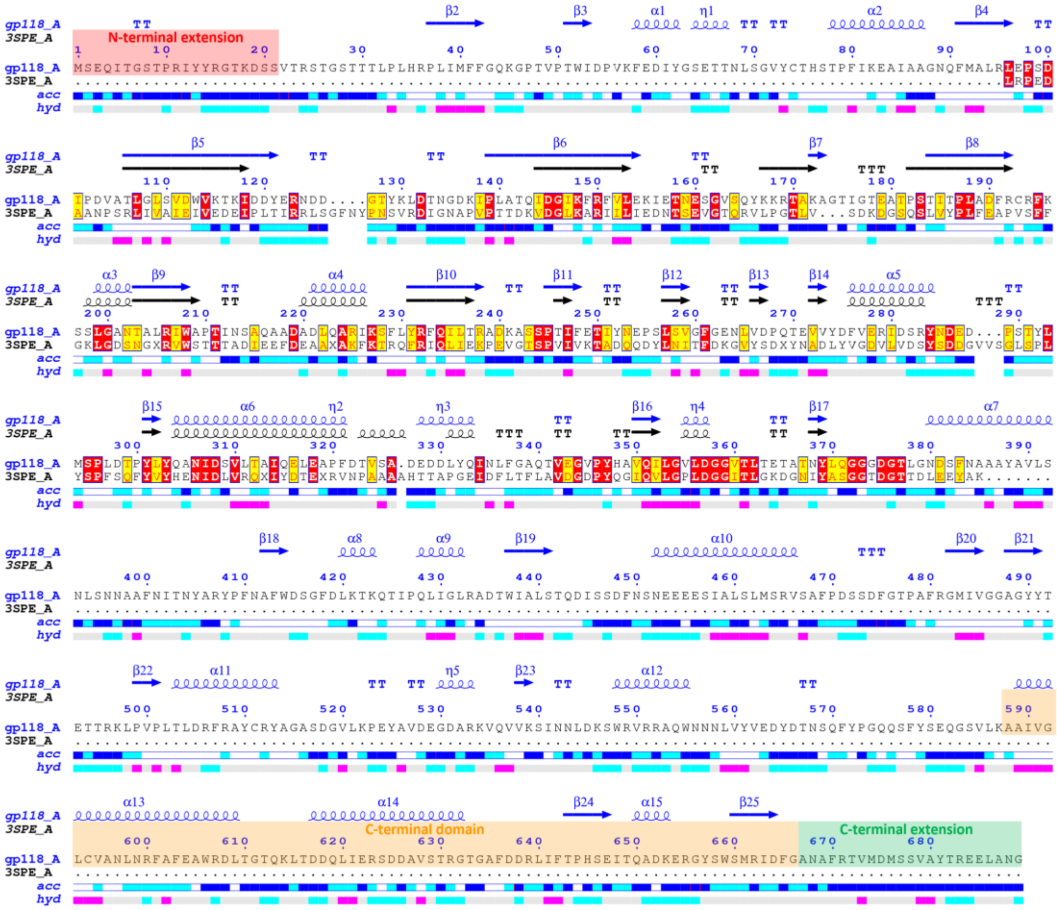

Supplementary Fig. 15 | The amino acid sequence of gp118 and alignment of gp29PR. The secondary-structure assignments of gp118 are indicated above the corresponding sequences. The amino acid sequence of the outer sheath component is L96 to G379. Using ENDscript 3, the tail sheath protein gp29PR from bacteriophage phiKZ (PDB ID: 3SPE) was aligned with the outer sheath component. The regions of interaction between the sheaths are highlighted (N-terminal extension, red; C-terminal domain, orange; C-terminal extension, green).

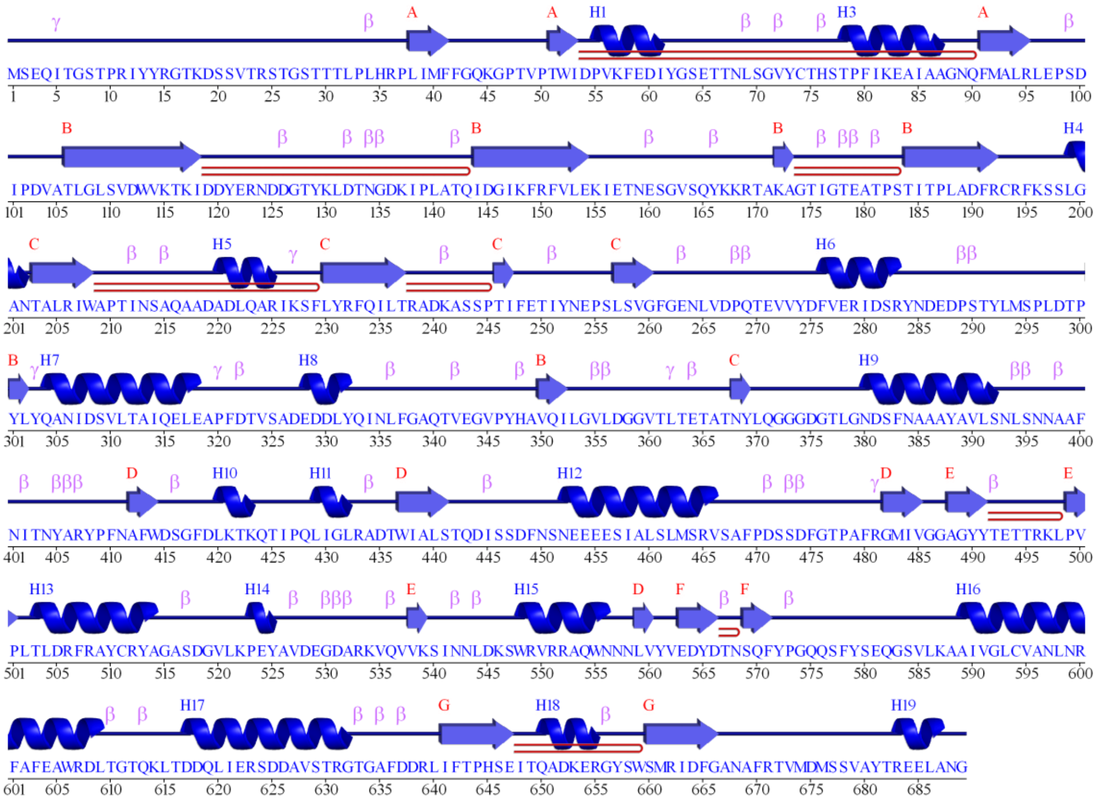

Supplementary Fig. 16 | The secondary structure diagrams of sheath protein gp118.

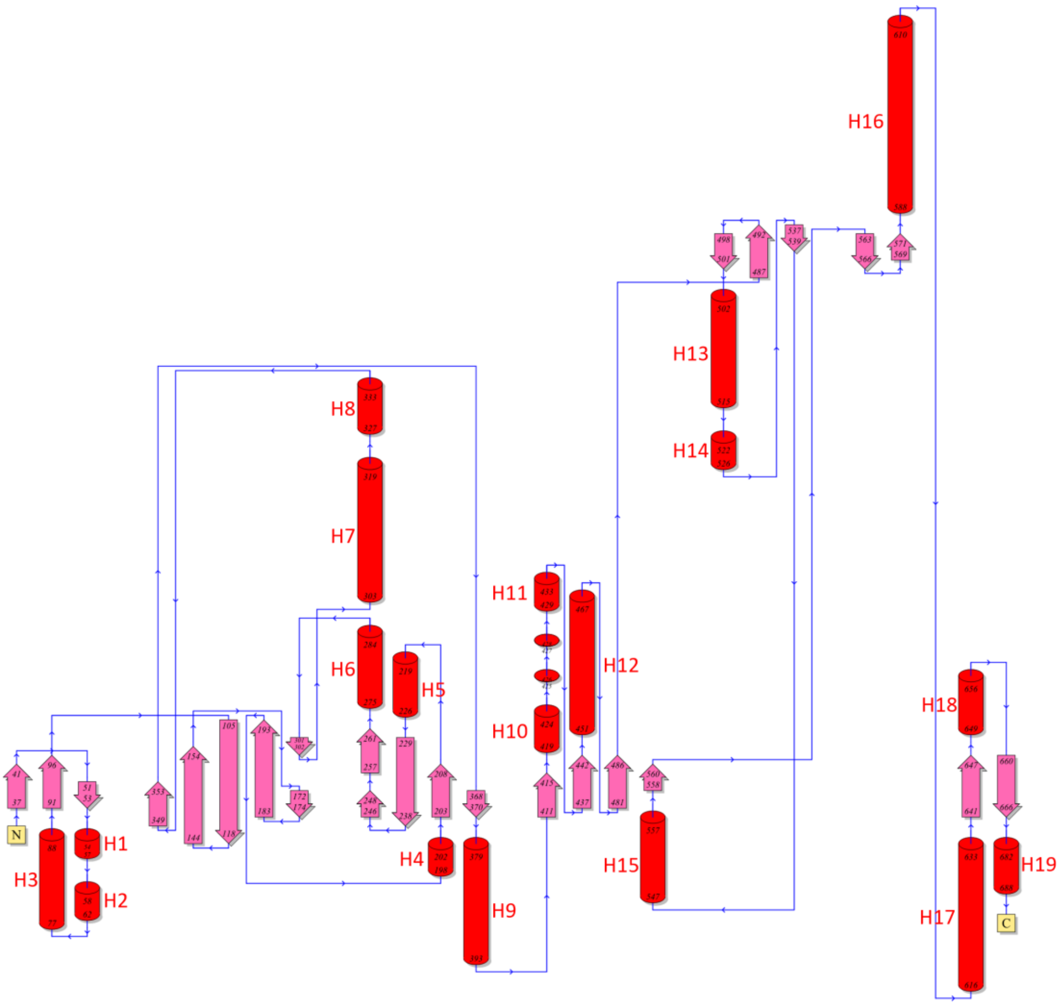

Supplementary Fig. 17 | The 2D secondary structure diagrams (topology) of sheath protein gp118.

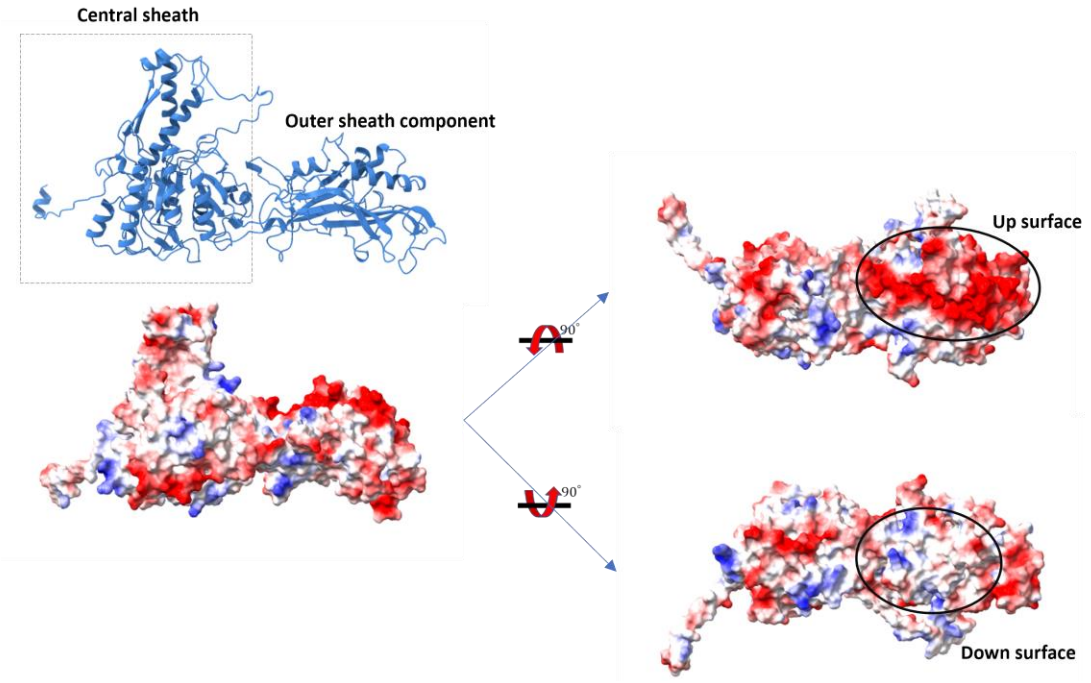

Supplementary Fig. 18 | The outer sheath component interactions. The outer sheath component interactions caused by electrostatic forces.

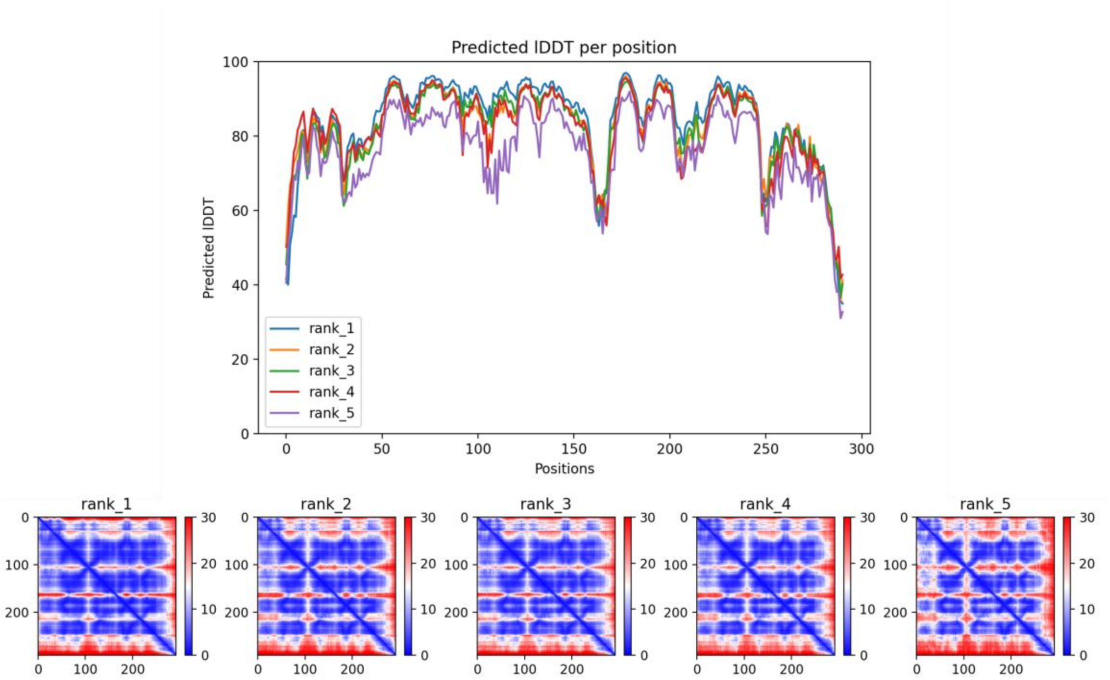

Supplementary Fig. 19 | The predicted lDDT of ϕKp24's inner tube protein gp119, generated from AlphaFold2. The model 1 was used for model building.

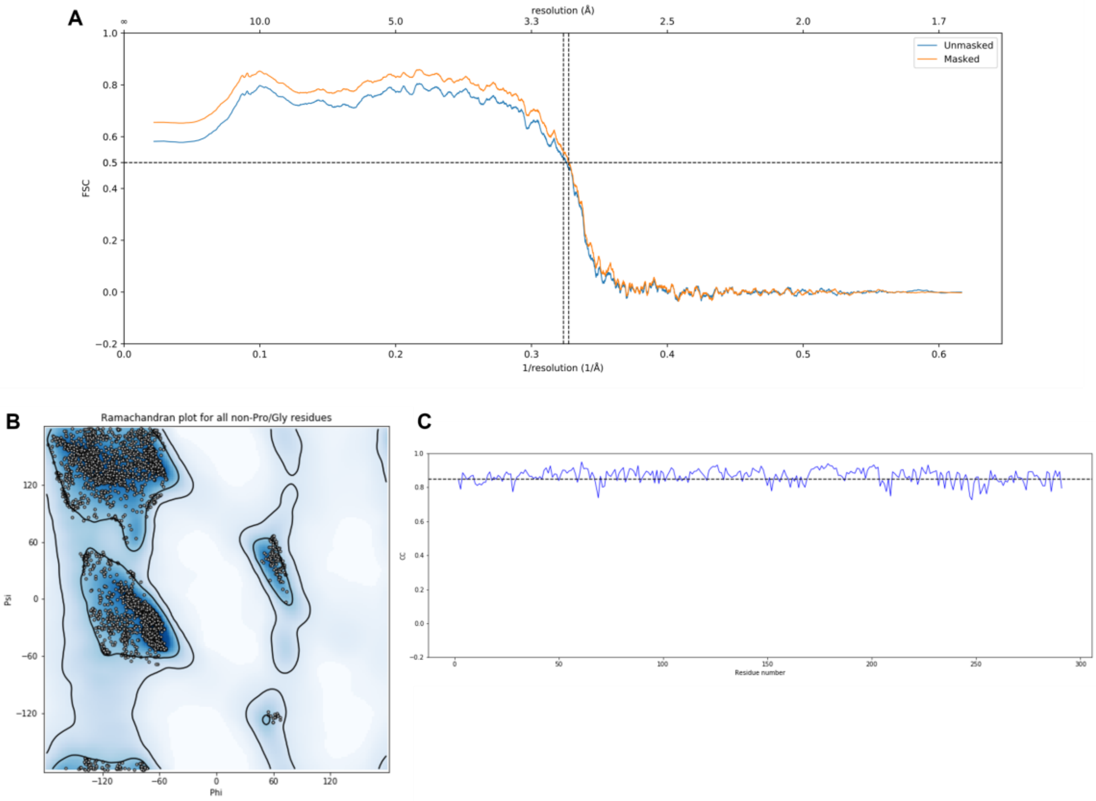

Supplementary Fig. 20 | Validation of the inner tube model (A) Model-map FSC curve of inner tube (3 discs, 18 monomers). **(B)** Ramachandran plot for all residues. **(C)** CC curve for all residues of one inner tube monomer gp119.

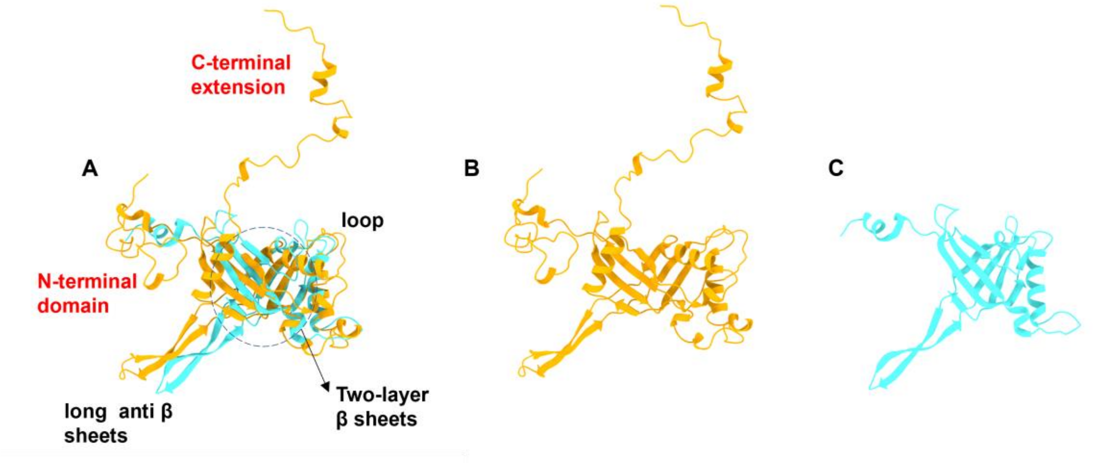

Supplementary Fig. 21 | Comparison of gp119 and T4's inner tube monomer (A) The ϕKp24's inner tube monomer gp119 (orange) compared with T4's inner tube monomer gp19 (cyan, PDB ID: 5W5F), using Matchmaker for alignment in ChimeraX. **(B)** A view of ϕKp24's inner tube monomer gp119 (orange). **(C)** A view of T4's inner tube monomer gp19.

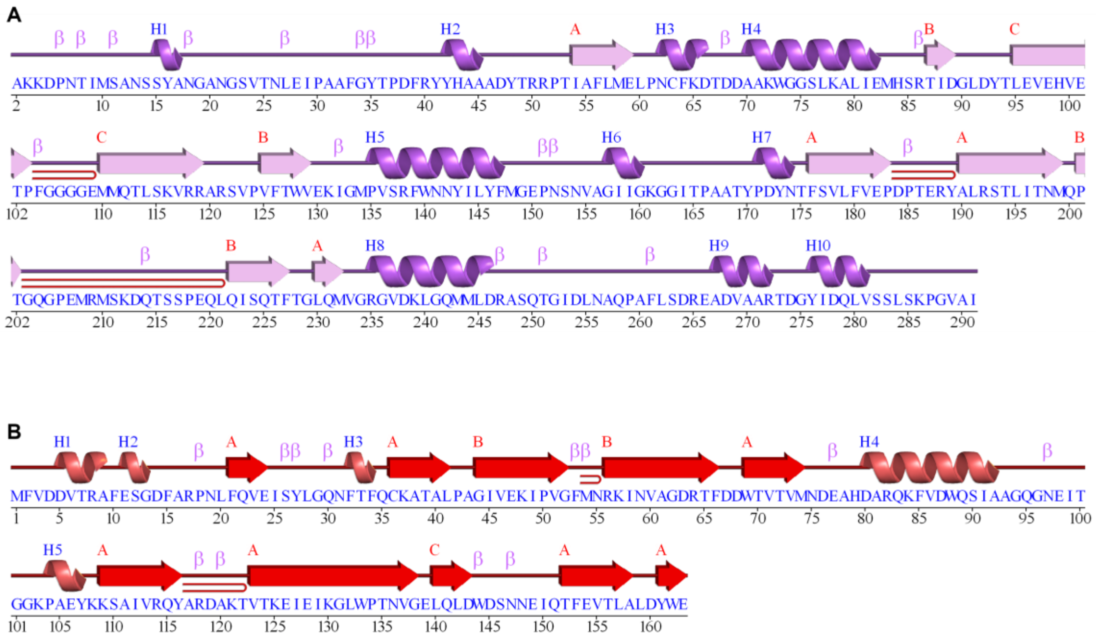

Supplementary Fig. 22 | The secondary structure diagrams of gp119 and T4's inner tube monomer **(A)** The diagram of phage ϕKp24's inner tube monomer gp119. **(B)** The diagram of phage T4's inner tube monomer gp19 (PDB ID: 5W5F).

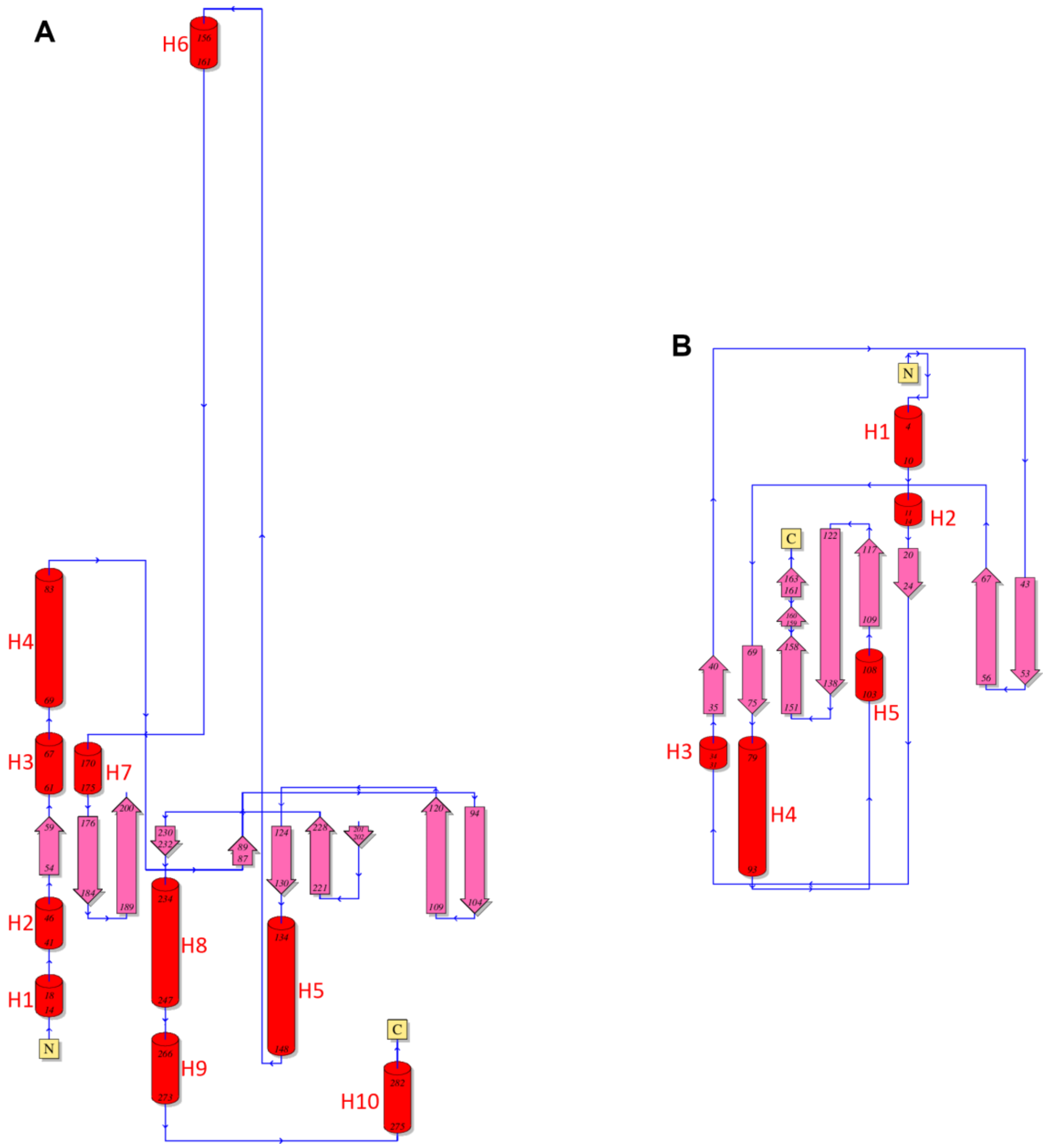

Supplementary Fig. 23 | The 2D secondary structure diagrams (topology) of gp119 and Phage T4‘s inner tube monomer (A) The diagram (topology) of phage ϕKp24’s inner tube monomer gp119. **(B)** The diagram (topology) of T4‘s inner tube monomer gp19.

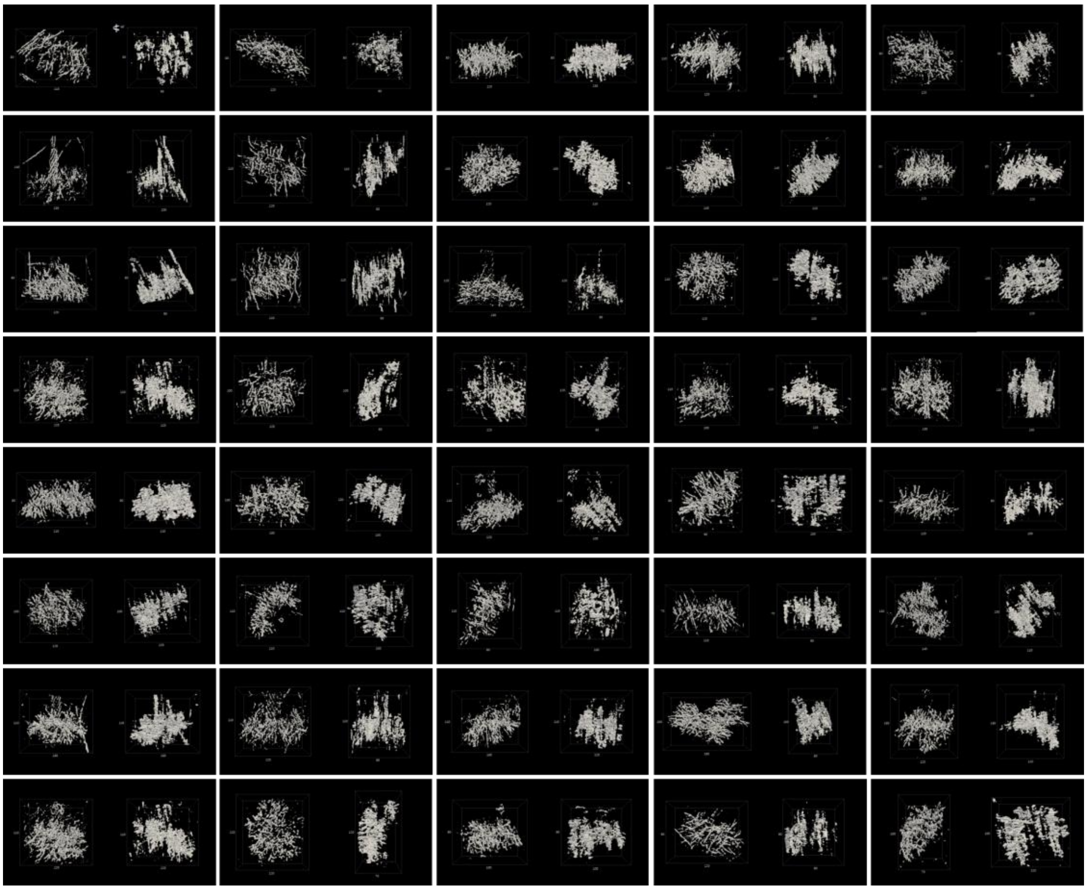

Supplementary Fig. 24 | 40 representative tail-fibers structures of pre-infection phages. 40 representative tail-fibers structures are selected from 89 tail-fibers structures of pre-infection phage. Each right image shows the side view of the left structure after rotation by 90˚. Both the outer box and red line are scale bar with pixel unit. Pixel size is 1.312 nm.

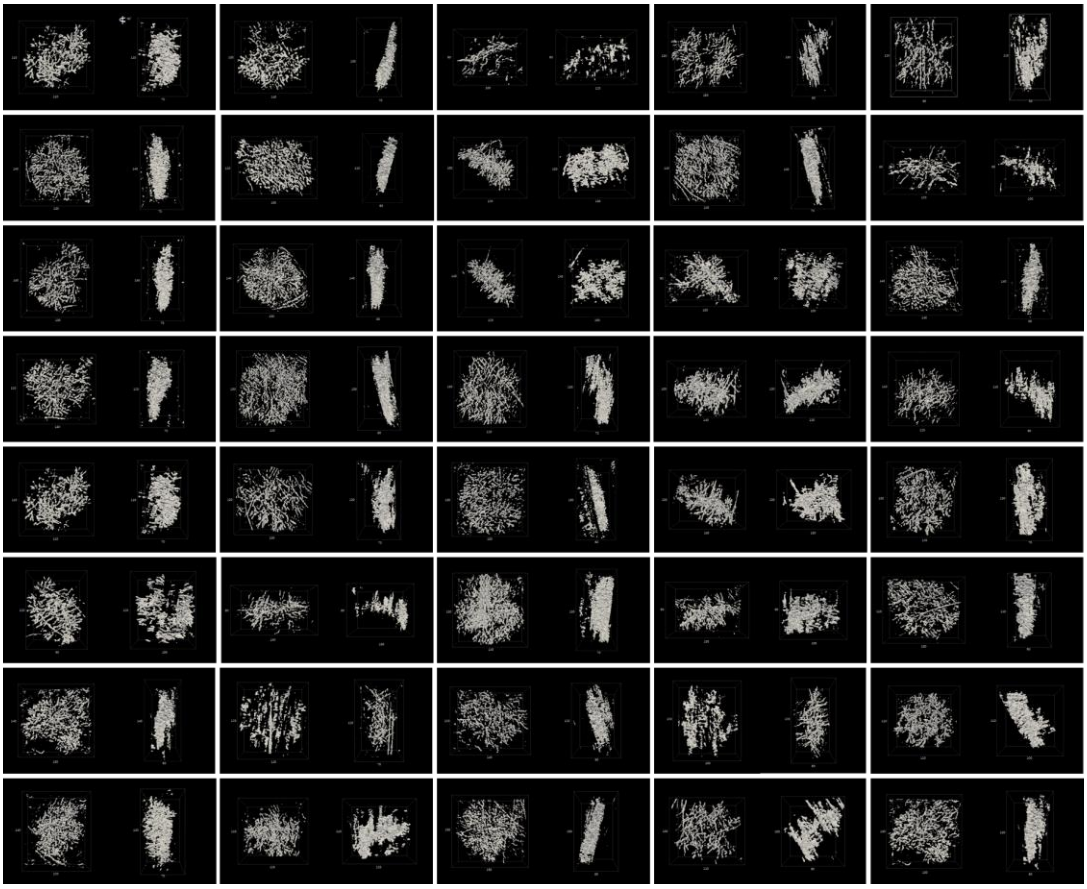

Supplementary Fig. 25 | 40 representative tail-fibers structures of post-infection phage attached to host. 40 representative tail-fibers structures are selected from 338 tail-fibers structures of post-infection phage. Each right image shows the side view of the left structure after rotation by 90˚. Both the outer box and red line are scale bar with pixel unit. Pixel size is 1.312 nm.

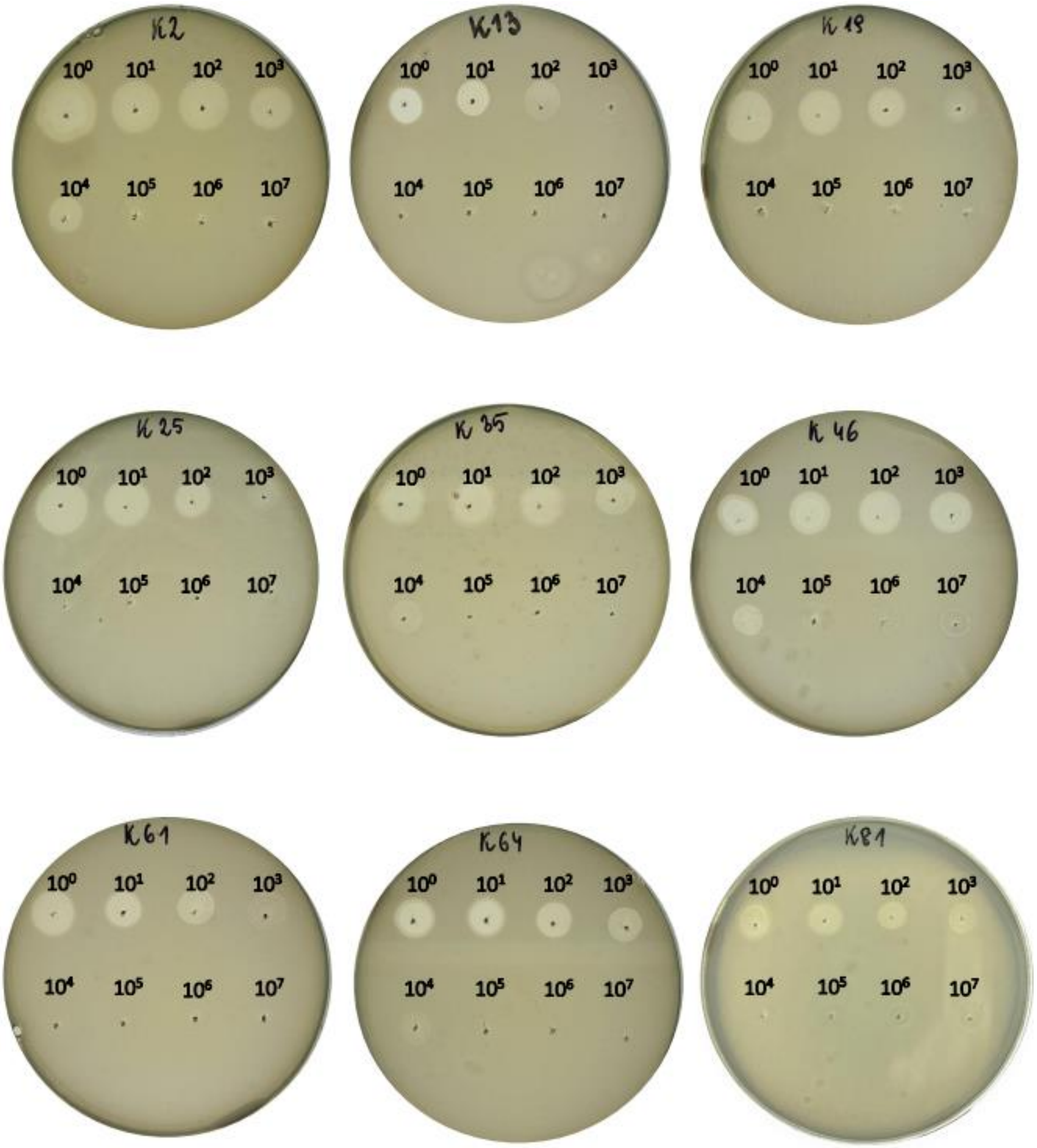

Supplementary Fig. 26 | A spot test of ϕKp24 phage on capsular serotypes of *K. pneumoniae*. 10 μl of each of a serial dilution (100-10-7) of the ϕKp24 phage suspension (107 pfu/mL) was spotted onto bacterial lawn. The presence of plaques and capsule depolymerase-generated halo zone were observed after overnight incubation. The bacteriophage ϕKp24 can infect 9 capsular serotypes of *K. pneumoniae* - K2 (CIP 52.145), K13 (CIP 52.217), K19 (CIP 52.223), K25 (CIP 52.230), K35 (CIP 53.10), K46 (CIP 53.22), K61 (NCTC 9181), K64 (NCTC 9184) and K81 (NCTC 11358).

	Phage ϕKp24 Empty Capsid	Phage ϕKp24 Full Capsid	Phage ϕKp24 Extended Tail
**Magnification**	64,000		
**Data collection / Final Pixel size (Å)**	0.685 / 2.055		0.685 / 1.37
**Voltage (kV)**	300		
**CS (mm)**	2.7		
**Detector**	Gatan K3		
**Detector mode**	SuperRes, Counted, non-CDS		
**Energy filter (eV)**	20		
**Electron exposure (e^-/Å^2)**	30		
**Frames**	30		
**Defocus Range (μm)**	-1 to -5		
**Number of movies collected**	34,229		
**Initial particle images (no.)**	290,280		114,132
**Final particle images (no.)**	19,193	8,869	81869
**Symmetry imposed**	I1		C6
**Inter-box distance (Å)**	/		56.97
**Nr. asym. units/box**	/		2
**Max. curvature**	/		0.05
**Helical rise (Å)**	/		39.03
**Helical twist (°)**	/		20.89
**Masked resolution at FSC=0.143 (Å)**	4.11	4.33	3.03
**Map sharpening B-factor (Å^2)**	-40	-45	-106
**EMDB**	EMD-13862	EMD-14356	EMD-14357

Supplementary Table. 1 | Cryo-EM data collection parameters and processing

	**Hexamers** (1central hexamer, 6 hexamers)	**Pentamers** (1 central pentamer, 5 hexamers)	**Outer sheath** (3 discs, 18 monomers)	**Inner tube** (3 discs, 18 monomers)
**PDB**	8BFL	8BFP	8AU1	8BFK
**Model compositions**				
Amino Acids	23940	19950	12402	5220
**Mean B factors (Å^2)**				
Amino Acids	93.66	81.39	67.82	38.58
**R.m.s. deviations**				
Bond lengths (Å)	0.004	0.004	0.005	0.03
Bond angles (˚)	0.846	0.846	0.629	0.531
**Validation**				
MolProbity score	2.09	2.08	1.58	1.35
Clash score	14.02	13.72	4.57	3.75
Rotamers outliers (%)	0.26	0.23	0.00	0.00
EMRinger score	1.43	1.54	4.59	4.98
**Ramachandran plot**				
Favored (%)	93.23	93.31	95.04	96.89
Allowed (%)	6.51	6.46	4.96	3.11
Outliers (%)	0.26	0.23	0.00	0.00
**Rama-Z**				
Whole	-1.15 (0.05)	-1.08 (0.06)	-1.21 (0.07)	-0.29 (0.12)
Helix	0.15 (0.08)	0.15 (0.09)	0.78 (0.10)	0.62 (0.18)
Sheet	0.26 (0.07)	0.24 (0.07)	-0.48 (0.11)	-0.50 (0.17)
Loop	-1.65 (0.05)	-1.52 (0.06)	-1.65 (0.07)	-0.28 (0.11)
**Model vs. Data**				
CC (mask)	0.78	0.81	0.83	0.86
CC (peaks)	0.69	0.62	0.76	0.67
CC (volume)	0.78	0.79	0.83	0.84

Supplementary Table. 2 | Model refinement and validation statistics
