# Supplementary material for: High-resolution reconstruction of a Jumbo-bacteriophage infecting capsulated bacteria using hyperbranched tail fibers
Source: Nat Commun. 2022 Nov 24;13:7241. doi: 10.1038/s41467-022-34972-5 (PMC9700779; doi:10.1038/s41467-022-34972-5)
Supplement: Supplementary file 3 — Description of Additional Supplementary Files [file 41467_2022_34972_MOESM3_ESM.pdf]

Description of Additional Supplementary Files

File Name: Supplementary Data 1
Description: Using the Klebsiella strains from K serotype collection to identify the specific capsule degrading enzymes produced by phage φKp24

File Name: Supplementary Data 2
Description: In silico prediction of phage φKp24 tail fibers domain structure

File Name: Supplementary Movie 1
Description: Animation of the capsid organization of φKp24

File Name: Supplementary Movie 2
Description: Animation of φKp24 attachment, tail fibers rearrangement and DNA ejection
