# Supplementary material for: High-resolution reconstruction of a Jumbo-bacteriophage infecting capsulated bacteria using hyperbranched tail fibers
Source: Nat Commun. 2022 Nov 24;13:7241. doi: 10.1038/s41467-022-34972-5 (PMC9700779; doi:10.1038/s41467-022-34972-5)
Supplement: Supplementary file 8 — Reporting Summary [file 41467_2022_34972_MOESM8_ESM.pdf]

nature portfolio

Corresponding author(s): Ariane Briegel
Last updated by author(s): Nov 3, 2022

Reporting Summary

Nature Portfolio wishes to improve the reproducibility of the work that we publish. This form provides structure for consistency and transparency in reporting. For further information on Nature Portfolio policies, see our Editorial Policies and the Editorial Policy Checklist.

Statistics

For all statistical analyses, confirm that the following items are present in the figure legend, table legend, main text, or Methods section.

n/a	Confirmed	
☐	☒	The exact sample size (*n*) for each experimental group/condition, given as a discrete number and unit of measurement
☐	☒	A statement on whether measurements were taken from distinct samples or whether the same sample was measured repeatedly
☒	☐	The statistical test(s) used AND whether they are one- or two-sided<br>*Only common tests should be described solely by name; describe more complex techniques in the Methods section.*
☒	☐	A description of all covariates tested
☒	☐	A description of any assumptions or corrections, such as tests of normality and adjustment for multiple comparisons
☐	☒	A full description of the statistical parameters including central tendency (e.g. means) or other basic estimates (e.g. regression coefficient) AND variation (e.g. standard deviation) or associated estimates of uncertainty (e.g. confidence intervals)
☒	☐	For null hypothesis testing, the test statistic (e.g. *F*, *t*, *r*) with confidence intervals, effect sizes, degrees of freedom and *P* value noted<br>*Give P values as exact values whenever suitable.*
☒	☐	For Bayesian analysis, information on the choice of priors and Markov chain Monte Carlo settings
☒	☐	For hierarchical and complex designs, identification of the appropriate level for tests and full reporting of outcomes
☒	☐	Estimates of effect sizes (e.g. Cohen's *d*, Pearson's *r*), indicating how they were calculated

Our web collection on statistics for biologists contains articles on many of the points above.

Software and code

Policy information about availability of computer code

Data collection	cryo-EM data was collected using Thermo Fisher Scientific EPU v2.8.1 (SPA) or SerialEM v3.9.1 (tomography)
Data analysis	Data was analyzed using IMOD v4.11.1; ResMap v1.1.4; Relion 3.1.2; MotionCorr2; gCTF v1.06; CTFFIND-4.1.18; UCSF Chimera v1.13 or v1.14; ChimeraX; RoseTTAFold; NAMD v2.13; VMD v1.9.4, Alphafold2, PHENIX version 1.19.2-4158, ISOLDE, Paraview,HI3D, ENDscript2, PDBsum, HMMER, Phyre2.<br>Code for neural network available under DOI 10.5281/zenodo.7277351

For manuscripts utilizing custom algorithms or software that are central to the research but not yet described in published literature, software must be made available to editors and reviewers. We strongly encourage code deposition in a community repository (e.g. GitHub). See the Nature Portfolio guidelines for submitting code & software for further information.

Data

Policy information about availability of data

All manuscripts must include a data availability statement. This statement should provide the following information, where applicable:

- Accession codes, unique identifiers, or web links for publicly available datasets
- A description of any restrictions on data availability
- For clinical datasets or third party data, please ensure that the statement adheres to our policy

The 3D cryo-EM maps generated in this study have been deposited at EMDB (the Electron Microscopy Data Bank, https://www.ebi.ac.uk/emdb/) with accession

code EMD-13862 for empty capsid of Klebsiella jumbo phage ϕKp24, code EMD-14356 for full capsid for Klebsiella jumbo phage ϕKp24, and code EMD-14357 for a length of the extended tail. Protein prediction data reported in this paper will be shared by the lead contact upon request. The atomic coordinates generated in this study have been deposited at wwPDB (Worldwide Protein Data Bank, http://www.wwpdb.org/) with accession PDB ID: 8BFL for hexamers of the empty capsid of Klebsiella jumbo phage ϕKp24, PDB ID: 8BFP for pentamer-hexamers of the empty capsid Klebsiella jumbo phage ϕKp24, PDB ID: 8AU1 for a length of outer sheath of the extended tail, and PDB ID: 8BFK for a length of inner tube of the extended tail.

Human research participants

Policy information about studies involving human research participants and Sex and Gender in Research.

Reporting on sex and gender	NA
Population characteristics	NA
Recruitment	NA
Ethics oversight	NA

Note that full information on the approval of the study protocol must also be provided in the manuscript.

Field-specific reporting

Please select the one below that is the best fit for your research. If you are not sure, read the appropriate sections before making your selection.

☒ Life sciences ☐ Behavioural & social sciences ☐ Ecological, evolutionary & environmental sciences

For a reference copy of the document with all sections, see nature.com/documents/nr-reporting-summary-flat.pdf

Life sciences study design

All studies must disclose on these points even when the disclosure is negative.

Sample size	Sample sizes for tomography was determined by counting the collected datasets. From these, we extracted the phage components that were detected by the neural network. More specifically, For the cryo-ET structural study of the tail fibers, we collected 89 tomograms. A subset of the data (338 tail fibers of post infection particles, 89 pre-infection particles) were used for structural comparison.<br>Sample size for SPA analysis was determined by the number of collected movies, and the particles that could be extracted from these data. More specifically, we collected 34229 individual movies. From this dataset we extracted 19193 particles (empty capsid), 8869 particles (full capsid) and 81869 (tail) to generate the maps.
Data exclusions	We excluded damaged or obstructed particles
Replication	NA (this is not applicable for structural studies. In the case of SPA, the density map is an average of thousands of particles. Replicates are not done for such experiments (see all previously published SPA maps in this journal and others). For tomography, we collected 89 tomograms and analyzed hundreds of particles as outlined above. Replication of data collection is not standard for cryo-electron tomography experiments.
Randomization	For tomography data, we assigned the phages into pre-infection particles if: (1) the capsid is full, (2) the tail is extended, (3) the phage is intact, and (4) the phage is not in contact with a bacterial cell. Similarly, we define the post-infection phages as follows: (1) the capsid is empty, (2) the tail is contracted, and (3) there are a few connections between the tips of fibers and the cell membrane.
Blinding	NA (no experiments performed which include study participants or similar). The study solely investigated bacterial samples and bacteriophages

Reporting for specific materials, systems and methods

We require information from authors about some types of materials, experimental systems and methods used in many studies. Here, indicate whether each material, system or method listed is relevant to your study. If you are not sure if a list item applies to your research, read the appropriate section before selecting a response.

Materials & experimental systems

n/a	Involved in the study
☒	☐ Antibodies
☒	☐ Eukaryotic cell lines
☒	☐ Palaeontology and archaeology
☒	☐ Animals and other organisms
☒	☐ Clinical data
☒	☐ Dual use research of concern

Methods

n/a	Involved in the study
☒	☐ ChIP-seq
☒	☐ Flow cytometry
☒	☐ MRI-based neuroimaging
